# Prevalence of Candida Species in the Oral Cavity of Patients Undergoing Head and Neck Radiotherapy

**DOI:** 10.5681/joddd.2009.020

**Published:** 2009-09-16

**Authors:** Arash Azizi, Masood Rezaei

**Affiliations:** ^1^Associate professor, Department of Oral Medicine, Faculty of Dentistry, Ahwaz Jundi Shapoor University of Medical Sciences, Ahvaz, Iran; ^2^Associate professor, Department of oral Medicine, Faculty of Dentistry, Tehran Azad University, Tehran, Iran

**Keywords:** Antifungal, Candida, radiotherapy, xerostomia

## Abstract

**Background and aims:**

Candidiasis is a common opportunistic infection in immunocompromised patients. Radiation to the head and neck affects the oral mucous membrane and produces xerostomia. Xerostomia alters the oral mucosa and predisposes them to colonization by Candida species. The aim of this study was evaluation of Candida species before and after radiotherapy.

**Materials and methods:**

Twenty patients undergoing radiation therapy were selected. None of the patients had taken any antibiotics and antifungals during the 3-month period prior to the study and did not take any during the study; in addition, they did not have any systemic conditions predisposing them to Candida infections. Swabs were collected from all the patients for Candida species culturing procedures 3±1 days before treatment and 2 and 4 weeks after radiotherapy. Swabs were inoculated on 2% Sabouraud’s dextrose agar. Different types of Candida species are specified by colony color. Analysis of variance was used to assess the difference between the periods before and after treatment.

**Results:**

Mean age of the patients were 59.4 years. Ten patients were Candida-positive before the initiation of radiotherapy. Eighteen and 20 patients were Candida-positive after two and four weeks of radiotherapy, respectively. The most frequent type of Candida in this study was Candida albicans both before and after radiotherapy.

**Conclusion:**

The present study suggests that patients undergoing head and neck radiotherapy should take antifungal agents, especially sugar-free agents, topical fluoride and salivary substitutes. The most commonly found Candida in this study was Candida albicans, which might be attributed to its high pathogenecity.

## Introduction

Candidiasis is a common opportunistic infection in immunocompromised patients. It manifests itself as both a superficial and a systemic infection. Candidiasis refers to a multiplicity of diseases caused by a yeastlike fungus, Candida, and is the most common oral fungal infection in humans. The organism is a unicellular yeast of the Cryptococcus family. Transformation from a state of commensalism to that of a pathogen by this organism is attributed to local and systemic factors. Xerostomia and chronic local irritants may alter the oral mucous membranes, predisposing them to colonization and invasion by Candida species. Shifts in the bacterial flora often accompany these situations and provide the opportunity for Candida species to multiply.^1^ Radiation to the head and neck region affects the oral mucous membranes and produces xerostomia.^2^ In irradiated patients the most common clinical infection of the oropharynx is candidiasis.^3 , 4^ Even though ionizing radiation is a common treatment modality for patients with head and neck cancer, and the most commonly found geni of Candida in saliva are albicans, tropicallis, and krusei, in the present study we investigated the prevalence of Candida species in the oral cavity of patients undergoing head and neck radiation.

## Materials and Methods

None of the patients had been taking antibiotics and antifungals during the 3-month period preceding the study or during the study. Swabs were collected from all the patients for Candida culture procedures at baseline, i.e. 3±1 days before radiotherapy and 2 and 4 weeks after the irradiation. The patients received a total dose of 6000 cGy up to 6600 cGy in daily fractions of 200 cGy. Swabs were collected from the dorsal surface of the tongue. Samples were inoculated on 2% agar Sabouraud’s dextrose (Becton Dickinson Microbiology Systems, Cockeysville, USA). After transporting samples to the Sabouraud’s culture media plates, the plates were placed in an incubator at 37°C. The number of yeast colonies was counted after a 24-hour incubation period. To isolate the yeasts, 50 ^mg ^/_L_ of chloramphenicol was added to the Sabouraud’s culture media to prevent bacterial growth. After retrieving the plates from the incubator, presence or absence of Candida species in the plates was evaluated. Then different types of Candida were specified by Candida colony color. Green colonies are characteristic of Candida albicans (Figure 1a). Blue colonies are characteristic of Candida tropicalis (Figure 1b) and pink colonies are characteristic of Candida krusei (Figure 1c).


Figure 1. Candida albicans colony (a); Candida tropicalis colony (b); Candida krusei colony (c).

**a:**
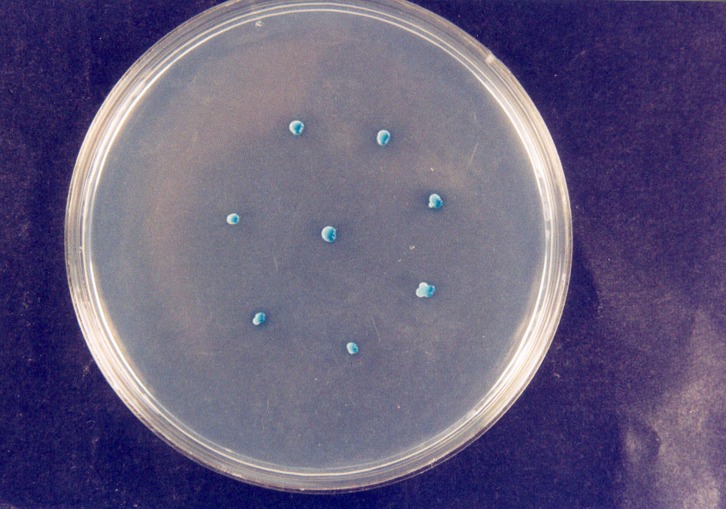

**b:**
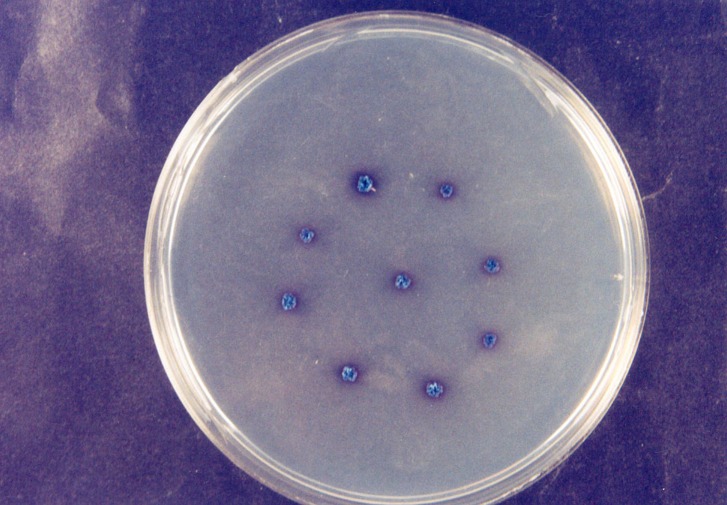

**c:**
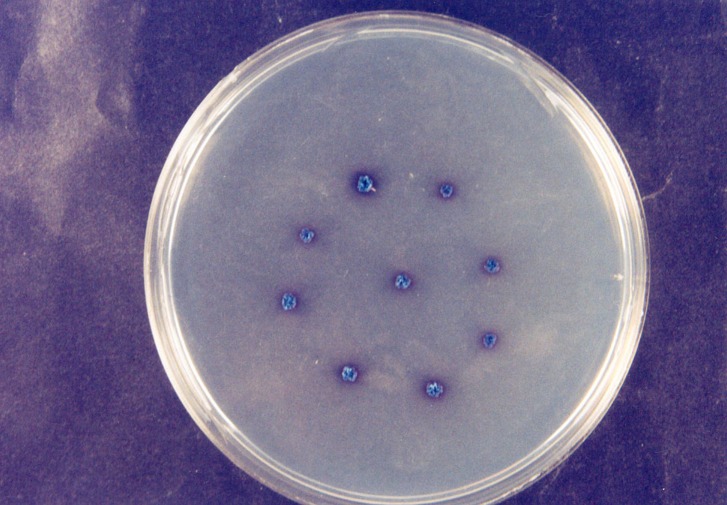

Analysis of variance was used to assess the differences between the results before and after the treatment procedure.

## Results

The particulars of the subjects are presented in Table 1. There were 20 patients with a mean age of 59.4 years. The group consisted of 16 men (mean age of 60.9 years) and 4 women (mean age of 57.9). All the subjects had undergone radiotherapy for their head and neck malignancies. The culture results of Candida were positive in 10 patients before the initiation of radiotherapy (Table 2). Two weeks after irradiation, swabs were taken again and Candida cultures were positive in 18 patients (Table 3). Four weeks after radiotherapy all the patients were Candida-positive (Table 4). Statistical analysis showed significant differences in Candida counts (P < 0.001) before and two weeks after radiotherapy; furthermore, there were significant differences in Candida counts before and after 4 weeks of radiotherapy (P < 0.000). However, there were no significant differences in Candida counts between 2- and 4-week intervals after treatment (P = 0.17).

**Table 1:** Demographic data of the patients

Sex	Number	Mean age	Age range
Men	14	60.9	44 – 75
Women	6	57.9	36 – 70
Total	20	59.4	36 - 75

**Table 2:** Prevalence of different types of Candida before radiotherapy

Candida types before radiotherapy	Number	Percentage
No Candida was detected (Negative )	10	50%
Candida albicans	7	35%
Candida tropicalis	2	10%
Candida krusei	1	5%
Total	20	100%

**Table 3:** Prevalence of different types of Candida after 2 weeks of radiotherapy

Candida types after 2 weeks of radiotherapy	Number	Percentage
Negative	2	10%
Candida albicans	12	60%
Candida albicans/ tropicalis	3	15%
Candida albicans/krusei	3	15%
Total	20	100%

**Table 4:** Prevalence of different types of Candida after 4 weeks of radiotherapy

Candida types after 4 weeks of radiotherapy	Number	Percentage
Negative	0	0
Candida albicans	13	65%
Candida albicans/ tropicalis	3	15%
Candida albicans/krusei	4	20%
Total	20	100%

## Discussion

Infections caused by Candida are on the rise as the number of immunocompromised patients in the community increases. Thus, oral candidiasis is the most common oral opportunistic infection in immunocompromised patients.^5^ Intensive chemotherapy, high doses of oral and systemic corticosteroids, potent antibiotics and underlying diseases such as diabetes mellitus, neutropenia, and xerostomia have all contributed to this phenomenon.^6^ External-beam radiation is a standard treatment modality for head and neck tumors, and the salivary glands are often within the field of radiation. Doses of ≥50 Gy will result in permanent salivary gland damage and symptoms of oral dryness. Acute detrimental effects on salivary function can be observed within a week after treatment is instituted at doses of approximately 2 Gy daily and patients will present with voice complaints as a result of oral dryness by the end of the second week. Typically, at doses >50 Gy, salivary dysfunction is severe and permanent.^2^ Speech problems, dysphagia, and increased dental caries are complaints that dramatically affect the quality of life for patients with radiation-induced salivary gland dysfunction. This patient population is susceptible to other oral complications, including candidaisis.^7^ Since patients undergoing radiation therapy have reduced salivary flow, they are susceptible to oral fungal and bacterial infections. An inverse correlation has been reported between salivary flow rate and Candida counts in saliva.^8 , 9^ Based on the results of the present study we believe that increase in Candida infections in patients undergoing head and neck radiotherapy is a result of decreases in salivary flow rates. Although other Candida species may be involved, Candida albicans is the major etiologic agent in oral and systemic candidiasis.^10^ The present study showed that strains of Candida albicans are more prevalent than other species of Candida before and after initiation of radiotherapy.

## Conclusion

Due to increased risk of Candida infections in patients undergoing head and neck radiotherapy we suggest that these patients use antifungal agents, especially sugar-free agents, during the treatment course. Vaginal clotrimazole troches and dissolved nystatin powder may be used orally as sugar-free antifungal agents. Since patients with xerostomia may have too little saliva to dissolve troches, antifungal oral rinses are preferred.^2^ Daily prescription-strength topical fluoride is recommended to help control caries. In addition, several symptomatic treatment modalities are available to replace saliva in these patients. Water is by far the most important factor. Patients should be encouraged to sip water throughout the day. This will help moisten the oral cavity, hydrate the mucosa, and clear debris from the mouth. There are many commercially available salivary substitutes, which patients can use to decrease caries rate and comfort the oral mucosa.^2^
